# Integration of prevention and control measures for female genital schistosomiasis, HIV and cervical cancer

**DOI:** 10.2471/BLT.20.252270

**Published:** 2020-07-06

**Authors:** Dirk Engels, Peter J Hotez, Camilla Ducker, Margaret Gyapong, Amaya L Bustinduy, William E Secor, Wendy Harrison, Sally Theobald, Rachael Thomson, Victoria Gamba, Makia C Masong, Patrick Lammie, Kreeneshni Govender, Pamela S Mbabazi, Mwelecele N Malecela

**Affiliations:** a Uniting to Combat NTDs, Chemin de la Gouille 8, 1291 Commugny, Switzerland.; b National School of Tropical Medicine, Baylor College of Medicine, Houston, United States of America (USA).; c Schistosomiasis Control Initiative, London, England.; d Institute of Health Research, University of Health and Allied Sciences, Ho, Volta Region, Ghana.; e Clinical Research, London School of Hygiene and Tropical Medicine, London, England.; f Center for Global Health, Centers for Disease Control and Prevention, Atlanta, USA.; g International Public Health, Liverpool School of Tropical Medicine, Liverpool, England.; h Tropical Disease Biology, Liverpool School of Tropical Medicine, Liverpool, England.; i Obstetrics and Gynaecology, University of Nairobi, Nairobi, Kenya.; j Catholic University of Central Africa, Yaoundé, Cameroon.; k Neglected Tropical Diseases Support Center, The Task Force for Global Health, Decatur, USA.; l Human Rights and Gender, Joint United Nations Programme on HIV/AIDS, Geneva, Switzerland.; m Department of Control of Neglected Tropical Diseases, World Health Organization, Geneva, Switzerland.

## Abstract

Female genital schistosomiasis as a result of chronic infection with *Schistosoma haematobium* (commonly known as bilharzia) continues to be largely ignored by national and global health policy-makers. International attention for large-scale action against the disease focuses on whether it is a risk factor for the transmission of human immunodeficiency virus (HIV). Yet female genital schistosomiasis itself is linked to pain, bleeding and sub- or infertility, leading to social stigma, and is a common issue for women in schistosomiasis-endemic areas in sub-Saharan Africa. The disease should therefore be recognized as another component of a comprehensive health and human rights agenda for women and girls in Africa, alongside HIV and cervical cancer. Each of these three diseases has a targeted and proven preventive intervention: antiretroviral therapy and pre-exposure prophylaxis for HIV; human papilloma virus vaccine for cervical cancer; and praziquantel treatment for female genital schistosomiasis. We discuss how female genital schistosomiasis control can be integrated with HIV and cervical cancer care. Such a programme will be part of a broader framework of sexual and reproductive health and rights, women’s empowerment and social justice in Africa. Integrated approaches that join up multiple public health programmes have the potential to expand or create opportunities to reach more girls and women throughout their life course. We outline a pragmatic operational research agenda that has the potential to optimize joint implementation of a package of measures responding to the specific needs of girls and women.

## Résumé

La schistosomiase génitale féminine, résultant d'une infection chronique à *Schistosoma haematobium* (également connue sous le nom de bilharziose), continue d'être largement ignorée par les responsables des politiques de santé nationales et internationales. Si le monde lui accorde son attention en vue de mener une action à grande échelle contre la maladie, c'est surtout pour déterminer s'il s'agit d'un facteur de risque pour la transmission du virus de l'immunodéficience humaine (VIH). Pourtant, la schistosomiase génitale féminine est associée à des douleurs, des saignements et peut engendrer l'hypofertilité, voire la stérilité. Par conséquent, celles qui en souffrent sont souvent stigmatisées, et le problème est courant dans les régions endémiques d'Afrique subsaharienne. Cette maladie doit donc être considérée comme composante à part entière d'une approche globale de la santé et des droits humains pour les femmes et filles africaines, à l'instar du VIH et du cancer du col de l'utérus. Chacune de ces trois maladies fait l'objet d'une intervention préventive ciblée qui a déjà fait ses preuves: le traitement antirétroviral et la prophylaxie pré-exposition pour le VIH; le vaccin contre le papillomavirus humain pour le cancer du col de l'utérus; et l'administration de praziquantel pour la schistosomiase génitale féminine. Le présent document se penche sur la manière d'intégrer la schistosomiase génitale féminine dans la prise en charge du VIH et du cancer du col de l'utérus. Un tel programme fera partie d'un cadre plus vaste consacré aux droits et à la santé sexuelle et reproductive, à l'émancipation des femmes et à la justice sociale en Afrique. Les approches intégrées qui regroupent plusieurs programmes de santé publique permettent d'élargir des perspectives ou de créer des opportunités visant à atteindre un plus grand nombre de filles et de femmes tout au long de leur vie. Nous exposons les grandes lignes d'un programme de recherches pragmatiques et opérationnelles capable d'optimiser la mise en œuvre conjointe d'une série de mesures qui répondent aux besoins spécifiques des filles et des femmes.

## Resumen

Los responsables de formular las políticas sanitarias nacionales y globales siguen ignorando en gran medida la esquistosomiasis genital femenina como consecuencia de la infección crónica por *Schistosoma haematobium* (conocida comúnmente como bilharziasis). La atención internacional para adoptar medidas de gran alcance contra la enfermedad se centra en determinar si es un factor de riesgo para la transmisión del virus de la inmunodeficiencia humana (VIH). Sin embargo, la propia esquistosomiasis genital femenina está vinculada al dolor, las hemorragias y la infertilidad o subfertilidad, lo que conduce al estigma social, además de ser un problema común para las mujeres de las áreas en donde la esquistosomiasis es endémica en el África subsahariana. Por consiguiente, la enfermedad debe ser reconocida como otro componente de un programa integral de salud y de derechos humanos para las mujeres y las niñas de África, junto con el VIH y el cáncer de cuello uterino. Cada una de estas tres enfermedades tiene una intervención preventiva específica y comprobada: la terapia antirretroviral y la profilaxis previa a la exposición para el VIH; la vacuna contra el virus del papiloma humano para el cáncer de cuello uterino; y el tratamiento con praziquantel para la esquistosomiasis genital femenina. Se analiza cómo el control de la esquistosomiasis genital femenina se puede integrar con la atención del VIH y el cáncer de cuello uterino. Ese programa formará parte de un marco más amplio de salud y de derechos sexuales y reproductivos, de empoderamiento de la mujer y de justicia social en África. Los enfoques integrados que unen múltiples programas de salud pública tienen el potencial de ampliar o crear oportunidades para llegar a más niñas y mujeres a lo largo de sus vidas. Se describe a grandes rasgos un programa de investigación operacional pragmático que tiene el potencial de optimizar la implementación conjunta de una serie de medidas que respondan a las necesidades específicas de las niñas y de las mujeres.

## ملخص

إن داء البلهارسيا التناسلية الأنثوية كنتيجة للإصابة المزمنة بالبلهارسيا الدموية (المعروفة باسم البلهارسيا)، يستمر في تعرضه للتجاهل إلى حد كبير من جانب واضعي السياسات الصحية الوطنية والعالمية. يركز الاهتمام الدولي للعمل على نطاق واسع ضد المرض على ما إذا كان عامل خطر لانتقال فيروس نقص المناعة البشرية (HIV). إلا إن داء البلهارسيا التناسلية الأنثوية نفسه يرتبط بالألم والنزيف والعقم أو عدم الخصوبة، أو يؤدي إلى الوصم الاجتماعي، وهو مشكلة شائعة بالنسبة للنساء في المناطق الموبوءة بالبلهارسيا في الدول الأفريقية جنوب الصحراء الكبرى. وعلى ذلك يجب الاعتراف بالمرض كمكون آخر من مكونات جدول أعمال الصحة الشاملة وحقوق الإنسان للنساء والفتيات في أفريقيا، إلى جانب فيروس نقص المناعة البشرية ((HIV وسرطان عنق الرحم. كل من هذه الأمراض الثلاثة لها تدخل وقائي مستهدف ومثبت: وهو العلاج بمضادات الفيروسات القهقرية، والوقاية قبل التعرض لفيروس نقص المناعة البشرية/الإيدز؛ لقاح فيروس الورم الحليمي البشري لسرطان عنق الرحم؛ وعلاج البرازيكوانتيل لمرض البلهارسيا التناسلية الأنثوية. نحن نناقش كيف يمكن دمج السيطرة على داء البلهارسيا التناسلية الأنثوية، مع رعاية نقص المناعة البشرية المكتسبة/ الإيدز، وسرطان عنق الرحم. سيكون هذا البرنامج جزءاً من إطار عمل أوسع للصحة والحقوق الجنسية والإنجابية، وتمكين المرأة والعدالة الاجتماعية في أفريقيا. إن النهج المتكاملة التي تشمل العديد من برامج الصحة العامة، لديها القدرة على توسيع أو خلق فرص للوصول إلى المزيد من الفتيات والنساء طوال فترات حياتهن. نحن نوجز جدول أعمال بحثي تشغيلي عملي، لديه إمكانية تحسين التنفيذ المشترك لحزمة من التدابير التي تستجيب للاحتياجات المحددة للفتيات والنساء.

## 摘要

由于感染慢性埃及裂体吸虫（俗称血吸虫）而导致的女性生殖器血吸虫病，在很大程度上仍然被国家和全球卫生政策制定者所忽视。国际社会针对该疾病采取的大规模行动的关注焦点在于其是否为人体免疫缺陷病毒 (HIV) 传播的危险因素。然而，女性生殖器血吸虫病本身与伤痛、出血和生育能力低下或不孕症有关，进而导致患者蒙受社会耻辱，而且在撒哈拉以南非洲血吸虫病流行地区，这种疾病已成为常见问题。因此，应将该疾病与 HIV 和宫颈癌一起，认定为非洲妇女和女童全面健康和人权议程的另一个组成部分。这三种疾病中的每一种都有针对性和经过验证的预防性干预措施：针对 HIV 的抗逆转录病毒治疗和暴露前的预防措施；针对宫颈癌的人乳头瘤病毒疫苗；以及针对女性生殖器血吸虫病的吡喹酮治疗。我们讨论如何将女性生殖器血吸虫病的控制与 HIV和宫颈癌护理结合起来。此类方案将成为非洲性健康和生殖健康与权利、赋予妇女权力和社会正义这一宽泛框架的一部分。结合多种公共卫生方案的综合方法有可能扩大或创造机会，帮助更多女童和妇女终生受益。我们拟定了一项针对实际运作研究的议程，该议程有可能优化联合实施一揽子措施，以满足女童和妇女的特定需要。

## Резюме

Генитальный шистосомоз у женщин в результате хронической инфекции, вызванной паразитом *Schistosoma haematobium* (более распространенное название — бильгарция), по-прежнему обходят вниманием при принятии решений в сфере здравоохранения как на национальном, так и на мировом уровне. Международное внимание к крупномасштабным мероприятиям по борьбе с заболеванием сосредоточено вокруг вопроса, является ли бильгарциоз фактором риска при передаче вируса иммунодефицита человека (ВИЧ). Но и сам по себе шистосомоз у женщин связан с болью, кровотечениями, нарушениями фертильности или бесплодием, что ведет к социальной стигматизации, которая очень распространена среди женщин в регионах, эндемичных по шистосомозу (к югу от Сахары). Следовательно, данное заболевание следует рассматривать в качестве компонента комплексной системы по охране здоровья и прав человека у женщин и девочек в Африке наряду с ВИЧ и раком шейки матки. Для каждого из этих трех заболеваний существуют целенаправленные и надежные методы профилактики: антиретровирусная терапия и доконтактная профилактика для ВИЧ, вакцинация против вируса папилломы человека для рака шейки матки и лечение празиквантелом для женского генитального шистосомоза. Авторы обсуждают возможность интеграции мер по контролю заболеваемости женским генитальным шистосомозом в схему медицинской помощи при ВИЧ и раке шейки матки. Такая программа станет частью более широкой концепции сексуального и репродуктивного здоровья и прав человека, расширения прав и возможностей женщин и социальной справедливости в Африке. Интегрированный подход, объединяющий многочисленные программы общественного здравоохранения, может расширить или создать новые возможности для более широкого охвата женщин и девочек на протяжении их жизни. Авторы описывают практичный план операционных исследований, который может помочь в оптимизации совместного внедрения пакета мер, отвечающих конкретным потребностям девочек и женщин.

## Introduction

Gynaecological schistosomiasis as a result of chronic infection with *Schistosoma haematobium* (commonly known as bilharzia) has been described in the medical literature since the 1940s.^1^^,^^2^ In the early 1970s researchers described a syndrome that was close to what is known today as female genital schistosomiasis,^3^^,^^4^ a term first used in the mid-1990s.^5^ Female genital schistosomiasis is caused by an inflammatory reaction to schistosome eggs trapped in body tissue, leading to fibrosis and scarring of the female genital tract. Early signs of the disease are a burning sensation in the genitals, spot bleeding, abnormal discharge smell, bloody discharge, stress incontinence and lower abdominal pain.^6^ The disease can rapidly progress towards swellings or ulcerations of the vulva and vagina, causing genital bleeding, pain and dyspareunia.^7^ The symptoms can gradually evolve towards reproductive organ damage, characterized by sub- or infertility, ectopic pregnancy, spontaneous abortion, premature birth and low birth weight. The disease can therefore have serious sexual and reproductive health consequences for women in schistosomiasis-endemic areas, occurring primarily in poor populations in sub-Saharan Africa. Here we report on how female genital schistosomiasis has emerged as a concern, alongside HIV and cervical cancer, among the sexual and reproductive health issues affecting African girls and women.

## Knowledge and gaps

Female genital schistosomiasis has not yet been reviewed or assessed as a specific entity, nor as part of the burden of schistosomiasis, by the Global Burden of Diseases Study.^8^ Obtaining accurate disease burden information for the disease is daunting, given that the clinical awareness among health-care workers is low, and its diagnosis is both cumbersome and complex. Additionally, the disease is not mentioned in most medical textbooks, or in the lay media, which further compounds the very low awareness and poor diagnosis of the condition, including among health workers.^9^ As a result, the disease has been largely ignored by national and global health policy-makers.^10^

A standard diagnosis of female genital schistosomiasis requires colposcopy and histopathology and is often only conducted in well-equipped facilities that are not widely available in endemic areas. Community-based assessment of the disease burden is therefore challenging. Studies investigating the disease have been carried out in 18 countries in Africa, with a cumulative total of only 10 332 women examined (Bustinduy A, London School of Hygiene and Tropical Medicine, September 2019, personal communication). In Nigeria the prevalence of female genital schistosomiasis in four schistosomiasis-endemic communities was 27.4% based on self-reported symptoms (87 of 317 women tested) and 70.0% as confirmed by gynaecological examination (14 women in a subsample of 20).^11^ Detection of *S. haematobium* deoxyribonucleic acid in vaginal lavage has a good positive predictive value for genital lesions, especially during the earlier stages of female genital schistosomiasis (70% overall, 77% in young women below the age of 25 years).^12^ The method offers diagnostic opportunities for further research and evaluation projects. In contrast, schistosome egg detection in urine, the gold standard diagnosis for urinary schistosomiasis, has a correlation of only 30% with the presence of genital lesions.^12^

A recent study in Zambia aimed to validate community-based diagnosis of female genital schistosomiasis by genital self-swabs. The prevalence of active schistosomiasis was 5.5% (33 out of 603 women) as detected by urine microscopy and 15.1% (91 out of 601 women) by circulating anodic antigen testing.^13^ However, 25.6% (135) of 527 women tested were found to have female genital schistosomiasis as diagnosed by agreement among two imaging experts and a computer-assisted image analysis (Bustinduy A, Bilharzia and HIV Study, London School of Hygiene and Tropical Medicine, September 2019, personal communication). Urine-based assessment of the occurrence of schistosomiasis in a community is not therefore a reliable proxy for the level of female genital schistosomiasis.

Female genital schistosomiasis has been suspected as a risk factor for the transmission of human immunodeficiency virus (HIV) for more than 25 years, based on pathophysiological, immunological and epidemiological data.^14^^,^^15^ However, only in 2006 was an association between female genital schistosomiasis and HIV first reported based on field data.^16^ Since then, the scientific documentation of urogenital schistosomiasis as a potential risk factor for HIV has been strengthened.^17^^–^^22^ This evidence needs to be considered along with the strong possibility that lesions accompanying female genital schistosomiasis are potential entry points for HIV. Unfortunately, conducting randomized controlled trials or prospective studies may require withholding essential medicines for either schistosomiasis or HIV prevention and treatment. Ethical concerns therefore make it virtually impossible to design studies looking for a causal association. Most data on schistosomiasis and HIV interactions therefore come from retrospective or observational studies that examine existing sera sets. These gaps in research have resulted in mixed designs on mixed schistosome species with a mixture of outcomes that have precluded consensus on whether schistosomiasis affects HIV infection. Retrospective serological studies are limited due to the difficulty of determining a woman’s genital schistosomiasis status or level of exposure to either schistosomiasis or HIV. Furthermore, few cross-sectional studies have focused specifically on female genital schistosomiasis because of the complex diagnostic procedures that are largely unsuitable in young adolescent girls. It is thus important to recognize that most studies of schistosomiasis–HIV co-infection do not specifically address female genital schistosomiasis. Nonetheless, the retrospective evidence from past studies, coupled with strong biological plausibility, are suggestive of a link between female genital schistosomiasis and increased risks of HIV acquisition.^9^

While the link between HIV and human papilloma virus (HPV) has been the subject of a systematic review and meta-analysis,^23^ the evidence linking female genital schistosomiasis with HPV infection and cervical cancer is less robust. Studies have found altered cervical mucosal gene expression and lower HPV- and cancer-protective interleukin-15 levels in women with *S. haematobium* infection and female genital schistosomiasis.^24^^,^^25^ This evidence, coupled with a strong geographical overlap of these three conditions, suggests potential links among them.

At the local level, within communities and the health workforce, there are gaps in knowledge and understanding about female genital schistosomiasis prevention, treatment and control. Health-care providers can easily confuse the symptoms of the disease with those of sexually transmitted infections or cervical cancer. Incorrect diagnosis and management can have profound psychosocial implications for pre-sexually active girls. The effect of the disease on fertility and pregnancy may have serious consequences for a woman’s relationships with her family and community, often resulting in marginalization, stigma, isolation and the threat of gender-based violence.^26^ In specialized gynaecological or obstetric clinics, characteristic female genital schistosomiasis lesions may be noticed, but not attended to because of the narrow disease focus of the clinic and limited clinical awareness among health workers.

## Rationale for an integrated approach

Female genital schistosomiasis is a serious gynaecological condition linked to pain, bleeding and severe social stigma in Africa. The consequences of the disease merit an urgent response in the broader sexual and reproductive health and rights agenda of girls and women in sub-Saharan Africa.^27^

Women and girls face multiple and intersecting health, social, gender and economic challenges, especially in the poorest and most marginalized and fragile parts of our world. Many, if not most of these women and girls in sub-Saharan Africa carry a triple burden of vulnerability to HIV, HPV/cervical cancer and female genital schistosomiasis. There is mounting evidence for a three-way interaction between these diseases and that controlling one may decrease the risk of unwanted outcomes for the two others.^17^^–^^25^ Each of the diseases has a targeted and proven preventive intervention: antiretroviral therapy and pre-exposure prophylaxis for HIV; the HPV vaccine for cervical cancer; and praziquantel treatment for female genital schistosomiasis. It is therefore logical and practical to break down the disease-specific approaches and aim for programmatic integration, using existing opportunities to reach girls and women throughout their life course. The benefits of such an integrated approach should be straightforward to document. Yet global health policy-makers, implementing partners and operational research initiatives still mostly separate female genital schistosomiasis from both HIV and cervical cancer prevention and treatment or care efforts. This lack of action happens despite the largely similar demographics of the women and girls affected by the three diseases and is a missed opportunity to galvanize action and expand access to prevention and treatment.

### High-level meeting

A high-level meeting was held to break the deadlock to large-scale implementation of female genital schistosomiasis interventions and to facilitate an integrated approach with HIV and cervical cancer prevention and care. Over a hundred international experts gathered on the side-lines of the 11th European Congress on Tropical Medicine and International Health in September 2019.^28^ The group called for immediate action to address this important and neglected sexual and reproductive health and rights issue affecting the lives of millions of sub-Saharan African women and girls. A need was identified to strengthen primary health care to promote access to praziquantel for girls and women at risk of schistosomiasis infection. The group also reiterated the demand for the full integration of interventions for female genital schistosomiasis with HIV and HPV/cervical cancer prevention and control in a comprehensive sexual and reproductive health package. As a result, the meeting focused on advancing the implementation science and strongly encouraged national researchers to take charge of operational research agendas for integrated delivery. The meeting also looked at efforts to address the social determinants of health and improve access to safe water and sanitation to reduce contact with infested waters for all those women at current or future risk.

### Operational research to facilitate implementation

The operational research needs that were highlighted during the meeting included: (i) community-level burden assessment of female genital schistosomiasis; (ii) strategies for ensuring regular praziquantel treatment of women and girls outside of school-based deworming programmes; (iii) clinical treatment protocols for existing female genital schistosomiasis; (iv) strategies to improve awareness of female genital schistosomiasis and community engagement with the issue; and (v) impact evaluation and economic analysis of integrated strategies versus separate, parallel approaches.

#### Community-level burden assessment

The Bilharzia and HIV study is currently evaluating genital self-swabbing as a potential low-cost approach to community-based diagnosis for female genital schistosomiasis in Zambia.^13^ While this approach offers enhanced opportunities for research and impact evaluation studies, it is unlikely to trigger a large-scale investigation of the female genital schistosomiasis burden. We can ask: is such a global or regional burden assessment necessary? At the high-level meeting,^28^ voices from experts working in the field confirmed that the public health relevance of the disease is easily revealed by its occurrence in gynaecological practice (Gamba V, University of Nairobi, 2019, personal communication) or qualitative methods in communities (Masong M, Catholic University of Central Africa, 2019, personal communication). Such information can be combined with capacity-building of the health workforce in diagnosing female genital schistosomiasis and the wider use of data platforms in local health services, including health centres equipped to test and treat cervical cancer. If so, the information should provide enough evidence to trigger programmatic integration. Key to the success of such a primary health-care approach is the establishment of a clear case definition of female genital schistosomiasis and the validation of diagnostic algorithms that need to be distributed for use in front-line health services.

#### Strategies for ensuring regular treatment

Chronic female genital schistosomiasis lesions can be prevented by regular treatment with praziquantel started at an early age, such as through primary school-based deworming programmes,^29^ and continued in later life at all available opportunities. Encouragingly, in addition to praziquantel for school-age children (donated free of charge in sub-Saharan Africa by Merck KGaA, Darmstadt, Germany), access to the treatment has recently been extended to selected groups of adults. There are also ongoing efforts to make a paediatric formulation widely available to preschool children. Yet, further operational research is needed to optimize opportunities for large-scale treatment of women and girls beyond deworming in school health programmes; for ensuring regular treatment of girls and women with praziquantel through the primary health-care system; or for test-and-treat approaches during early childhood or from adolescence onwards. An important component of this work includes better ways to diagnose schistosomiasis and female genital schistosomiasis in sexual and reproductive health and rights programmes. The urine dipstick for microscopic haematuria is currently the best proxy for diagnosis of urinary schistosomiasis. However, more sensitive rapid diagnostic tests based on antigen detection in urine are under development and will facilitate test-and-treat approaches in children younger than 5 years, adolescent girls and young women. Combined packages of rapid diagnostic tests and treatment could be tailored for use in health services and programmes. Clinical diagnosis of female genital schistosomiasis would be improved if links could be established with health-care facilities that provide cervical cancer screening programmes. While the aim of the current paper is to focus on female genital schistosomiasis, these principles are valid too for male genital schistosomiasis.^30^

#### Clinical treatment protocols

While its preventive effect is well documented, it is less clear whether praziquantel treatment at the standard dose improves established genital disease. In the Bilharzia and HIV Study in Zambia of women aged 18–31 years, the frequency of six female genital schistosomiasis-related symptoms all declined by 5 to 10 percentage points after a single dose (Bustinduy A, London School of Hygiene and Tropical Medicine, 2019, personal communication). This finding is consistent with the partial resolution of lesions observed in Malawi,^31^ South Africa^32^ and Zimbabwe^33^ after standard praziquantel treatment. However, we need additional research to determine the optimal clinical treatment of female genital schistosomiasis, potentially with higher and more prolonged doses of praziquantel, with or without additional anti-inflammatory drugs. The results will inform the revision of treatment guidelines and curricula in training institutions.

#### Strategies to improve awareness

Box 1 presents several questions surrounding the perception of female genital schistosomiasis as a prevailing sexual and reproductive health and rights problem.

Box 1 **Questions surrounding the perception of female genital schistosomiasis as a prevailing sexual and reproductive health and rights problem**

- How can we mobilize and engage communities, health services and their workforce so that female genital schistosomiasis is understood and acted upon appropriately and stigma is avoided?
- What are the enabling factors and barriers for the integration of female genital schistosomiasis, HIV and HPV/cervical cancer in sexual and reproductive health and rights programmes at the community level?
- How can we rapidly build the capacity of the skilled health workforce, front-line health workers and school teachers to become agents of change in favour of fair and comprehensive sexual and reproductive health and rights programmes?
- How can we increase community awareness of female genital schistosomiasis prevention, treatment and control and meaningfully engage sexual and reproductive health and rights and HIV activists?
- How can community activists become advocates within their communities and agents of demand for inclusive, non-stigmatizing, integrated and comprehensive services?
- How can community awareness and demand creation influence policy changes to address the issues of safe water and behavioural change to prevent schistosomiasis?^34^^,^^35^

HIV: human immunodeficiency virus; HPV: human papilloma virus.

All these are operational research questions that biomedical and social science researchers will need to jointly answer. Many lessons can be drawn from the experience of HIV with regards to mobilizing and engaging communities, amplifying the call for integrated services and creating demand for service and commodities to address the specific needs of women and girls when and where they need them.^36^

#### Impact evaluation and economic analysis

More studies to strengthen the evidence base for a cause-and-effect relationship among female genital schistosomiasis, HIV and HPV/cervical cancer would clearly be useful. Randomized controlled trial evidence would be ideal, but proven and well-established interventions are available for each of these diseases and cannot be withheld in a trial setting. Although research on neglected tropical diseases remains relatively underfunded, the lack of robust evidence should not be a deterrent to moving forward. Close monitoring and evaluation of the impact of a comprehensive response on all three diseases could potentially be nested into ongoing population-based impact assessment and evaluation efforts. The data should provide the answer to the question of whether implementation of the packages of care provides enhanced benefits, should be scaled up and offers opportunities for economies of scale.

## Three diseases: one integrated response

**Fig. 1:**
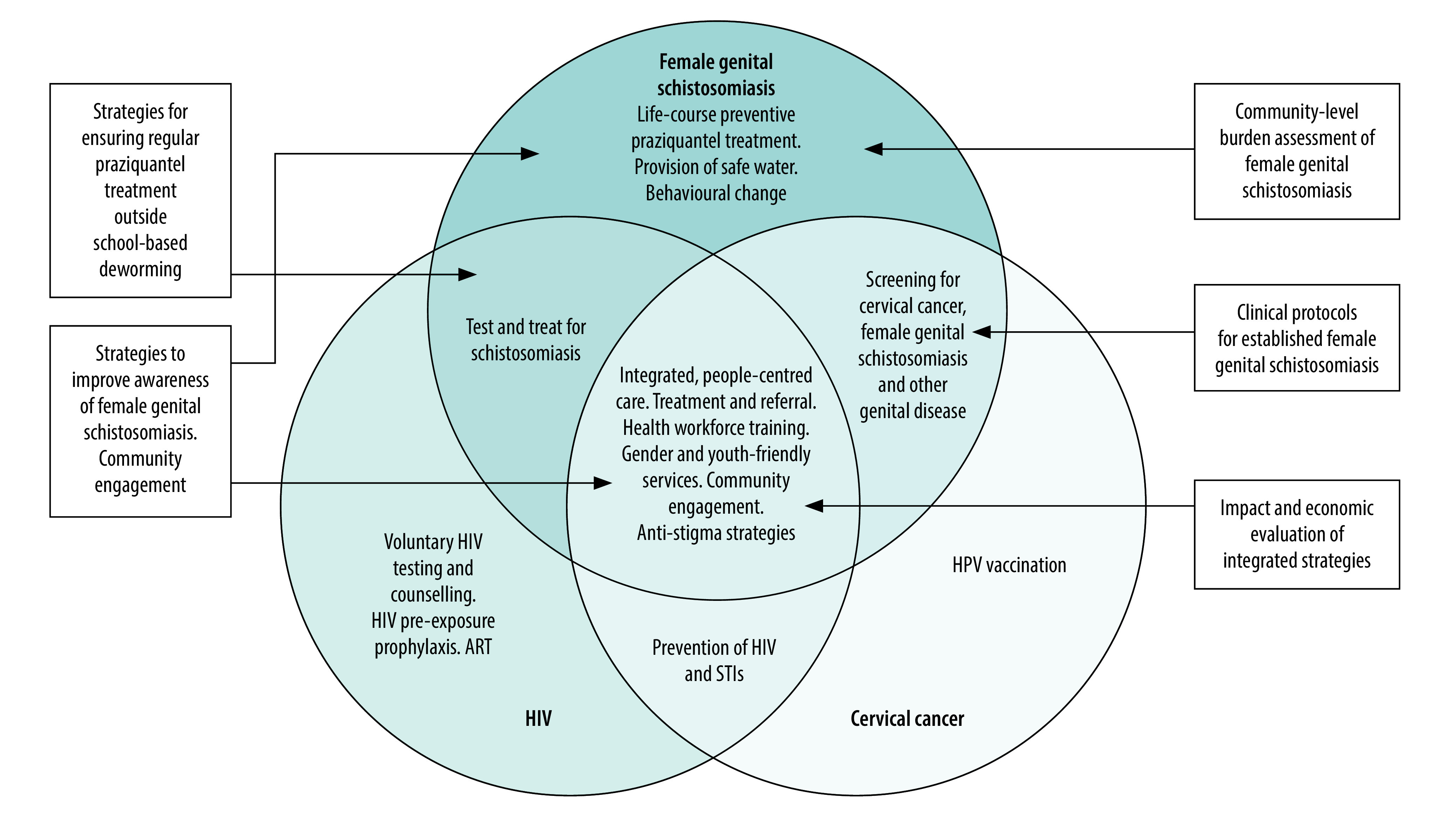
Conceptual framework for the integrated programmatic implementation of female genital schistosomiasis, HIV and HPV/cervical cancer

ART: antiretroviral therapy; HIV: human immunodeficiency virus; HPV: human papilloma virus; STIs: sexually transmitted infections.

Note: Boxes indicate where operational research goals will create opportunities for further improvement or evidence-building.

The physical and psychosocial impact of female genital schistosomiasis, along with the potential links across the three priority genital diseases, position female genital schistosomiasis as a pillar in a comprehensive sexual and reproductive health and rights agenda for women and girls in Africa.^37^ Despite the many unanswered questions, integrated action with the currently available tools and practices is highly needed and possible. We have summarized a conceptual framework for the integrated programmatic implementation of the three diseases (Fig. 1). The framework can be further tailored to include screening, treatment or referral for other common female genital diseases or complications of other infections, such as genital tuberculosis.

Although female genital schistosomiasis is preventable by regular treatment, too often praziquantel is not available within the primary health-care system outside of school-based, large-scale treatment.^38^ Many girls and women who do not attend schools are therefore missed. An integrated response to female genital schistosomiasis, HIV and cervical cancer is likely to boost access to praziquantel treatment for girls and women beyond primary schooling.

Established female genital schistosomiasis can be diagnosed during screening for cervical cancer, such as colposcopy and visual inspection of the cervix with acetic acid. The World Health Organization (WHO) has produced a visual aid to facilitate recognition of female genital schistosomiasis by clinical health-care professionals.^39^ The pocket atlas also allows inclusion of the disease in guidelines and training modules for the syndromic management of sexually transmitted infections and encourages parallel screening for HIV, sexually transmitted infections and cervical cancer.^40^ Furthermore, recognizing the need for an integrated response, the WHO and the Joint United Nations Programme on HIV/AIDS (UNAIDS) developed a joint advocacy brief in 2019. The document aims to increase knowledge and awareness of female genital schistosomiasis among policy-makers and affected communities, encouraging programmatic integration in areas endemic for urogenital schistosomiasis.^41^ The joint advocacy brief is similar to the 2016 UNAIDS advocacy brief on HIV and HPV cervical cancer.^42^ This earlier document has successfully mobilized civil society, notably sexual and reproductive health and rights activists and women living with HIV, to engage in and influence the WHO initiative to eliminate cervical cancer as a public health problem.

Preventive interventions for HIV, cervical cancer and female genital schistosomiasis can all be successfully delivered through existing programmes. An integrated programme would strengthen health systems and make a compelling case for a strategic change towards comprehensive sexual and reproductive health and rights services in affected regions (Table 1). A pragmatic operational research agenda has the potential to optimize joint implementation. As improved tools and practices are developed, sustained access to them will be needed in resource-poor environments. In this respect, we can draw lessons from the various models that have been developed and implemented to ensure access to medicines and diagnostics for neglected tropical diseases.

**Table 1:** Programmatic integration of the prevention and treatment of HIV, sexually transmitted infections, cervical cancer and female genital schistosomiasis in regions endemic for *Schistosoma haematobium*

Life stage	Target programmes	HIV and sexually transmitted infection interventions	Schistosomiasis and female genital schistosomiasis interventions	HPV and cervical cancer interventions	Community mobilization, education and counselling
Infants and young children (< 5 years)	Ante-, peri- and postnatal care. Mother and child health clinics. Immunization, services and campaigns. Preventive care through the integrated management of childhood illnesses.	Voluntary HIV testing services for mothers and children.	Test-and-treat for urinary schistosomiasis for mothers and children (dipstick urinalysis for microhaematuria or other).^a^ Treatment with praziquantel of mothers and children positive for *Schistosoma haematobium*.^b^ Systematic praziquantel treatment in highly endemic areas.	NA	Counselling for mothers on HIV prevention. Promotion of behavioural change for prevention and treatment of schistosomiasis. Topics to include safe bathing practices for infants and children. Awareness-building and information on HIV, schistosomiasis, female genital schistosomiasis and HPV/cervical cancer. Dialogue with mothers and caregivers about signs and symptoms of female genital schistosomiasis, sexually transmitted infections and cervical cancer. Referral to appropriate services if indicated. Community-based outreach, including generation of demand for accessible treatment and for improved water and sanitation services. Examples include: through community health clubs and community water, sanitation and hygiene management groups
Primary school-age children	School health programmes. School meal programmes. Programmes targeting or including children not enrolled in school.	NA	Regular treatment with praziquantel as part of deworming programmes. Frequency according to level of endemicity and WHO recommendations. Possible extension of deworming to siblings and non-enrolled school-age children in same communities. Safe water and (girl-friendly and inclusive) toilets in school.	HPV vaccination. Possible extension of HPV vaccination to include siblings and non-enrolled school-age children in recommended age range in same communities.	Education about schistosomiasis, communicable diseases or other tropical diseases in the area. Age-appropriate, comprehensive health, sexual and reproductive health and rights and life-skills education. Topics to include: HIV, female genital schistosomiasis and cervical cancer. Hygiene education, including menstrual hygiene for girls.
Adolescent girls (12–19 years)	Secondary school health programmes. Innovative programmes targeting youth and adolescents, both in and out of school. Should include migrant and vulnerable populations.^c^	Voluntary HIV testing services as appropriate. Refer to health services for further care if indicated.	Discussion about girl’s risk of schistosomiasis. Test-and-treat for urinary schistosomiasis if indicated. Alternatively: regular, context-specific large-scale treatment in areas highly endemic for schistosomiasis.	Catch-up HPV vaccination as appropriate.	Provide youth-friendly, gender-aware and age-appropriate comprehensive sexual and reproductive health and rights education. Topics to include: HIV, sexually transmitted infections, female genital schistosomiasis and cervical cancer. Referral to appropriate services if indicated. Hygiene education, including menstrual hygiene for girls. Age-appropriate comprehensive sexuality education. Topics to include: counselling on condom use, negotiating skills relating to sexual interactions and safe-sex practices. Community-based outreach, including generation of demand for comprehensive sexual and reproductive health and rights services.
Women (≥ 20 years)	Prenatal care and mother and child health programmes. Family planning. HIV screening and prevention programmes. Sexual and reproductive health clinics. Other programmes targeting women of reproductive age.	Offer voluntary HIV testing services. Refer to health services for further care if indicated. Evaluate additional risks and whether pre-exposure prophylaxis against HIV is indicated.	Discussion about woman’s risk of schistosomiasis. Test-and-treat for urinary schistosomiasis if indicated. Evaluate additional risk of female genital schistosomiasis. Refer to health services or cervical cancer clinics for further screening and care if indicated. For women with infertility, refer for screening and treatment of female genital schistosomiasis if indicated.	Promote regular cervical cancer screening and colposcopy in appropriate age-groups. Provide and facilitate access to cervical cancer screening services. Include screening and treatment for female genital schistosomiasis in cervical cancer screening services.	Provide information on symptoms and risks of HIV infection, sexually transmitted infections and female genital schistosomiasis. Query women about signs and symptoms of female genital schistosomiasis, sexually transmitted infections and cervical cancer. Facilitate referral to appropriate services if indicated. Hygiene education, including menstrual hygiene for women. Train physicians to begin colposcopy for women at younger ages and to recognize, diagnose and treat female genital schistosomiasis. Community-based outreach, engagement and generation of demand for comprehensive sexual and reproductive health and rights services in existing community-based structures. Examples include: women’s groups, mother and child health clinics and village health clubs.

HIV: human immunodeficiency virus; HPV: human papilloma virus; NA: not applicable; WHO: World Health Organization.

^a^ Antigen-based urine dipstick is under development.

^b^ Paediatric formulation of praziquantel is under development.

^c^ Examples include the Adolescents and Youth Program^43^ and the DREAMS partnership.^44^

Note: Adapted from WHO and Joint United Nations Programme on HIV/AIDS, 2019.^41^

## Conclusion

Several relevant global initiatives are already in place: the sustainable development goals, including universal health coverage;^36^ the 2016 United Nations political declaration on HIV;^45^ the HIV prevention 2020 road map^46^ and the H6 partnership to advance the Every Woman Every Child global strategy.^47^ The current global health environment offers opportunities to combine disease-specific initiatives; strengthen health systems at all levels to provide integrated, comprehensive and quality services; and to address the multifaceted and intersecting health, sociocultural, gender and economic issues facing women and girls. Integrated approaches, which have a strong rights-based approach, and which join up multiple public health programmes, create new opportunities and expand existing ways to reach more girls and women throughout their life span. In addition, an integrated approach provides opportunities to mobilize new resources and use existing resources more effectively. Building on lessons learnt from the response to the HIV epidemic, we need to expand and diversify partnerships beyond the traditional biomedical public health communities to engage advocates for sexual and reproductive health rights and women’s rights. Such expanded partnerships will help to position comprehensive prevention and control of female genital schistosomiasis, HIV and cervical cancer within the broader sexual and reproductive health and rights, women’s empowerment and social justice framework.
